# MicroRNA (MiR)-301a-3p regulates the proliferation of esophageal squamous cells via targeting PTEN

**DOI:** 10.1080/21655979.2020.1814658

**Published:** 2020-09-24

**Authors:** Nan Zhang, Jun Feng Liu

**Affiliations:** Department of Thoracic Surgery, The Fourth Hospital of Hebei Medical University, Shijiazhuang, China

**Keywords:** MiR-301a-3p, esophageal squamous cell carcinoma, PTEN, BCL-2, BAX

## Abstract

Human esophageal carcinoma (EC) is a common cancer, which leads to many deaths worldwide every year. Our study aimed to explore the mechanism of miR-301a-3p regulating the proliferation of esophageal squamous cell carcinoma (ESCC) cells. We had collected ESCC tissues and adjacent normal esophageal tissues from 47 patients. The relative levels of miR-301a-3p/U6 in ESCC tissues and cells were analyzed by real-time PCR. And we measured the relative protein levels of PTEN, BCL-2, BAX, and p-AKT/AKT by Western blot. Eca-109 cell proliferation was detected by MTT assay and colony formation. Compared with adjacent normal esophageal tissues, the relative level of miR-301a-3p/U6 was elevated in ESCC tissues. MiR-301a-3p could facilitate ESCC cell proliferation. And miR-301a-3p directly bind to PTEN 3ʹ-UTR and negatively regulated PTEN protein expression. Moreover, silencing PTEN could reversed inhibited proliferation of Eca-109 cells induced by miR-301a-3p inhibitor, while overexpression PTEN could reversed enhanced proliferation of Eca-109 cells induced by miR-301a-3p mimic. Taken together, miR-301a-3p promoted ESCC cell proliferation by supressing PTEN.

## Graphical abstract

**Figure:**
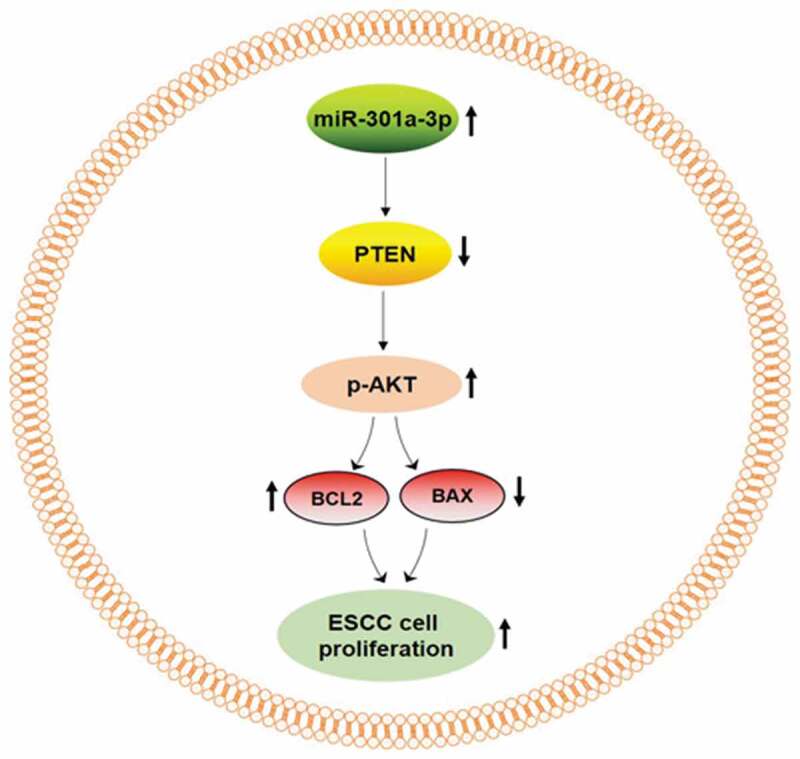

## Introduction

Esophageal carcinoma (EC) is a common cancer, which causes millions deaths worldwide every year [1]. ESCC and esophageal adenocarcinoma are two main pathological subtypes of EC. In China, most EC cases belong to ESCC [2]. The incidence rate of EC increases with age, which is rare in young people. Additionally, there are many risk factors leading to EC, such as gastroesophageal reflux disease, smoking, alcohol consumption and overweight [3]. The five-year survival rate of EC patients is still poor because many EC cases are diagnosed at an advanced stage. EC clinical presentations include weight loss, heartburn and dysphagia. The treatment of EC has been improved over the past decade, but it still needs to be improved [4].

MicroRNAs are highly conserved small noncoding RNAs, which can directly bind to target gene 3ʹ-UTR and inhibit protein expression [5]. The aberrant expression of microRNAs leads to various cancers [6,7]. MicroRNAs regulate the development of cancers, such as migration, apoptosis, proliferation and differentiation [8–10]. MiR-301a-3p expresses in many tissues, which belongs to miR-301 family. Aberrant expression of miR-301a-3p results in various cancers, such as pancreatic cancer [11], hepatocellular carcinoma [12] and laryngeal squamous cell carcinoma [13].

In this study, we had found the relative level of miR-301a-3p/U6 was increased in ESCC tissue and was correlated with tumor size and TNM stage. We aimed to determine the molecular mechanism of miR-301a-3p regulating ESCC cell proliferation. This study may provide a new therapeutic target for ESCC.

## Materials and methods

### Clinical samples

We had collected forty-seven pairs of ESCC tissues and adjacent normal esophageal tissues at the Fourth Hospital from 2016 to 2018. Forty-seven ESCC patients (age range 42–76 years, mean age of 65 years) were treated with esophagectomy and did not had any radiation treatment and chemotherapy drugs. This research was authorized by the Ethical Review Committee at the Fourth Hospital of Hebei Medical University. All patients in this study had signed the informed consent.

### Cell culture

Esophageal mucosal epithelial cell Het-1A and ESCC cell line Eca-109 were purchased from the National biomedical experimental cell resource bank (China) and were kept in RPMI-1640 medium including 10% FBS (Gibco, USA) and 1% penicillin-streptomycin (Solarbio, China) in a humidified atmosphere incubator (Thermo, USA) with a 5% CO2 at 37°C.

### MiR-301a-3p mimic and inhibitor transfection

Small RNA oligos, such as negative control mimic (NCm), miR-301a-3p mimic (301am), negative control inhibitor (NCi) and miR-301a-3p inhibitor (301ai) were purchased from GenePharma Company (China). HiperFect transfection reagent (Qiagen, Germany) was used to transfected small RNA oligos following by the product instruction.

### Western blot

Cell Lysis Buffer (Cell signaling Technology, USA) was used to extracted total protein sample of tissues and cells following by product instruction. And protein sample concentration was measured by BCA kit (Thermo, USA). 30 μg total protein was loaded in 10% SDS-PAGE gel and transferred to PVDF membrane (Millipore, USA). 8% nonfat milk was used to block PVDF membrane. And 1:1000 diluted primary antibodies were used to incubate PVDF membrane overnight at 4°C. Primary antibodies against PTEN (#9559), p-AKT (#4060) and AKT (#9272) and GAPDH (#5174) were purchased from Cell signaling Technology company. Primary antibodies against BCL2 (12789-1-AP) and BAX (50559-2-ig) were purchased from Proteintech company. Then the membrane was washed by TBST three times and incubated with 1:5000 diluted HRP-conjugated secondary antibody (Zhongshanjinqiao, China) for 2 hours at room temperature. Western blot bands were detected with ECL kit (Millipore, USA). And Image J was applied to analyze the band intensity.

### RNA extraction and real-time PCR

TRIzol reagent (Thermo, USA) was used to extract total RNA of tissues and cells following by the product instruction. For tissue specimens, 50 mg tissue sample was grinded by tissue homogenizer in 1.5 ml tube with 1 ml TRIzol reagent (Thermo, USA). For cell samples, cold PBS was used to wash 6-well plate three times. And 1 ml TRIzol reagent was added into each well of 6-well plate. 1 μg RNA was used as template to reversely transcribe into first-strand cDNA. Specific reverse transcription primers with a stem-loop tail for miR-301a-3p and U6 were described as Table 1. Then the relative level of miR-301a-3p was measured by real-time PCR by using SYBR Green kit (TaKaRa, Japan). We used U6 as a housekeeping gene to normalize the relative level of miR-301a-3p. And the relative level of miR-301a-3p/U6 was calculated by the 2-∆∆Ct method.

**Table 1.** The sequences of primers.

Primer	Sequences (5ʹ-3ʹ)
miR-301a-3p RT	5ʹ-GTCGTATCCAGTGCAGGGTCCGAGGTATTCGCACTGGATACGACGCTTTG-3’
U6 RT	5ʹ-GTCGTATCCAGTGCAGGGTCCGAGGTATTCGCACTGGATACGACAAAAATATG. −3’
miR-301a-3p F	5ʹ- GCGAGCAGTGCAATAGTATTGT-3’
U6 F	5ʹ- GCGCGTCGTGAAGCGTTC-3’
Reverse R	5ʹ- GTGCAGG GTCCGAGGT-3’

### Luciferase assay

A synthesized DNA fragment containing six repeats of binding site (Shanghai Sheng Gong, China) was ligated into pmirGLO vector (Promega, USA) to construct recombinant vector. PmirGLO vector and recombinant vector were transfected into Eca-109 cells for 48 h. And luciferase activity was measured with A dual-luciferase reporter assay system kit (Promega, USA).

### MTT assay

The day following the transfection of miR-301a-3p mimic or inhibitor, Eca-109 cells were digested by 0.25% trypsin and seeded in a 96-well cell culture plate with 2000 cells per well. MTT assay was performed at 24 hour, 48 hour, and 72 hour. First, added 20 μl 10 mg/ml MTT (Sigma, USA) into cell medium and incubated for 4 hours. Second, discarded all the supernatant in wells and added 150 μl DMSO (Sigma, USA). At last, shaked the plate and mixed well for 5 minutes. The absorbance at 490 nm was analyzed by Spectrophotometer (Bio-Rad, USA).

### Colony formation test

The day following the transfection of miR-301a-3p mimic or inhibitor, Eca-109 cells were digested by 0.25% trypsin and cultured in a 6-well plate with 300 cells per well for 2 weeks at 37°C. At fourteenth day, stain the cell colony by using crystal violet solution (2.5%) (Solarbio, China). The colonies that contained more than 50 cells were counted. The ratio of 301am to NCm or 301ai to NCi was calculated as the relative colony numbers.

### Statistical analysis

Statistical analysis was performed by SPSS 13.0 software (USA). All data are displayed as the mean ± SEM. The difference between two groups was compared by *t* test. And the difference between three or more groups was compared by one‑way ANOVA test followed by Tukey’s post-hoc test. And *p* values less than 0.05 was considered as significant difference. The correlation between the relative level of miR-301a-3p/U6 and the relative protein level of PTEN/GAPDH in tumor tissues was analyzed by Spearman correlation test.

### Results

#### The relative level of miR-301a-3p/U6 is elevated in ESCC tissues

**Figure 1.**
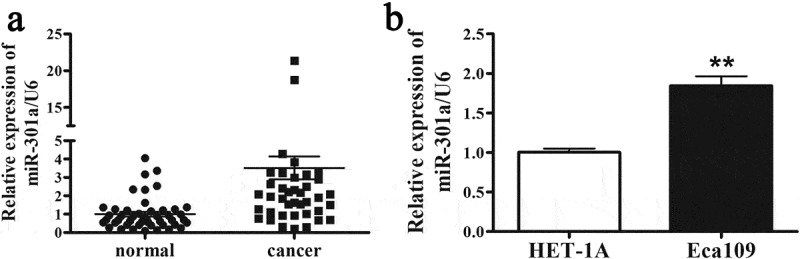
The relative level of miR-301a-3p is elevated in ESCC tissues. The relative level of miR-301a-3p/U6 is elevated in ESCC tissues (cancer group) compared with adjacent normal tissues (normal group) (a). And the relative level of miR-301a-3p/U6 in HET-1A and Eca-109 cells was also analyzed (b). **p < 0.01 vs normal group; n = 47 for ESCC tissues; **p < 0.01 vs HET-1A cell; n = 3.

To compare the relative level of miR-301a-3p/U6 in ESCC tissues and normal tissues, 47 pairs of normal tissues and ESCC tissues were collected and analyzed by real-time PCR. As Figure 1a indicated that the relative level of miR-301a-3p/U6 was dramatically upregulated by 3-fold in ESCC tissues. Furthermore, we tested the relative level of miR-301a-3p/U6 in Esophageal mucosal epithelial cell Het-1A and ESCC cell line Eca-109. In consistent with ESCC tissues, the relative level of miR-301a-3p/U6 was increased by about twofold in Eca-109 cells compared with Het-1A cells (Figure 1b). Next, we determined whether the relative level miR-301a-3p/U6 was associated with ESCC patient pathological characteristics. Table 2 indicated that the relative level of miR-301a-3p/U6 was significantly correlated with tumor size and TNM stage. Therefore, these results implied that increased miR-301a-3p expression might be related with the pathogenesis of ESCC.

**Table 2.** The correlation between the relative level of miR-301a-3p/U6 and pathological characteristics of ESCC patients (n = 47).

Clinicopathologic features	miR-301a-3p expression		p
	High (n = 14)	Low (n = 33)	
Gender			
Male	10	24	1
Female	4	9	
Age(years)			
>65	10	15	0.103
≤65	4	18	
Differentiation			
Well+moderate	8	25	0.163
Poor	6	8	
TNM stage			
I–II	5	25	0.009*
III–IV	9	8	
length			
≥4 cm	9	10	0.03*
<4 cm	5	23

#### MiR-301a-3p promotes ESCC cell proliferation

**Figure 2.**
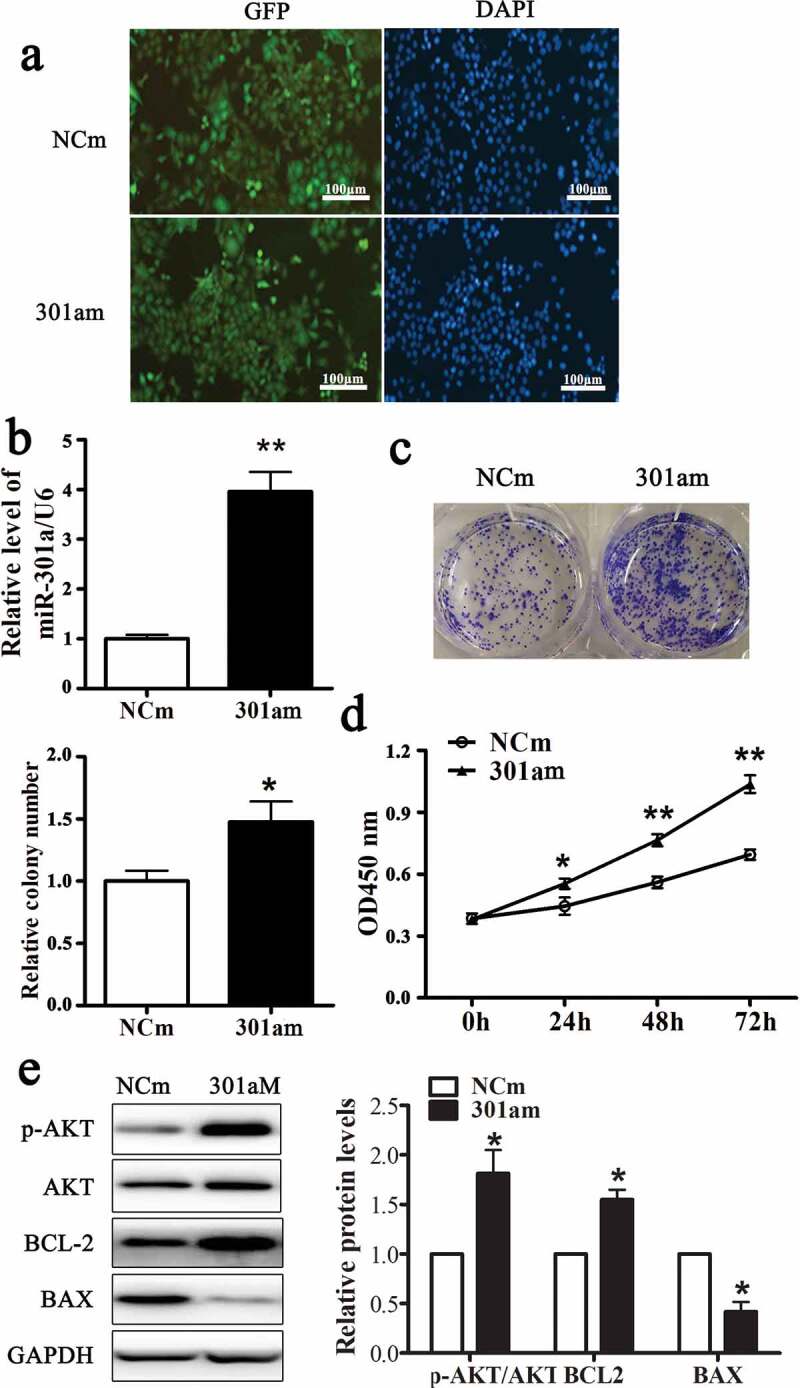
miR-301a-3p stimulates Eca-109 cell proliferation.

**Figure 3.**
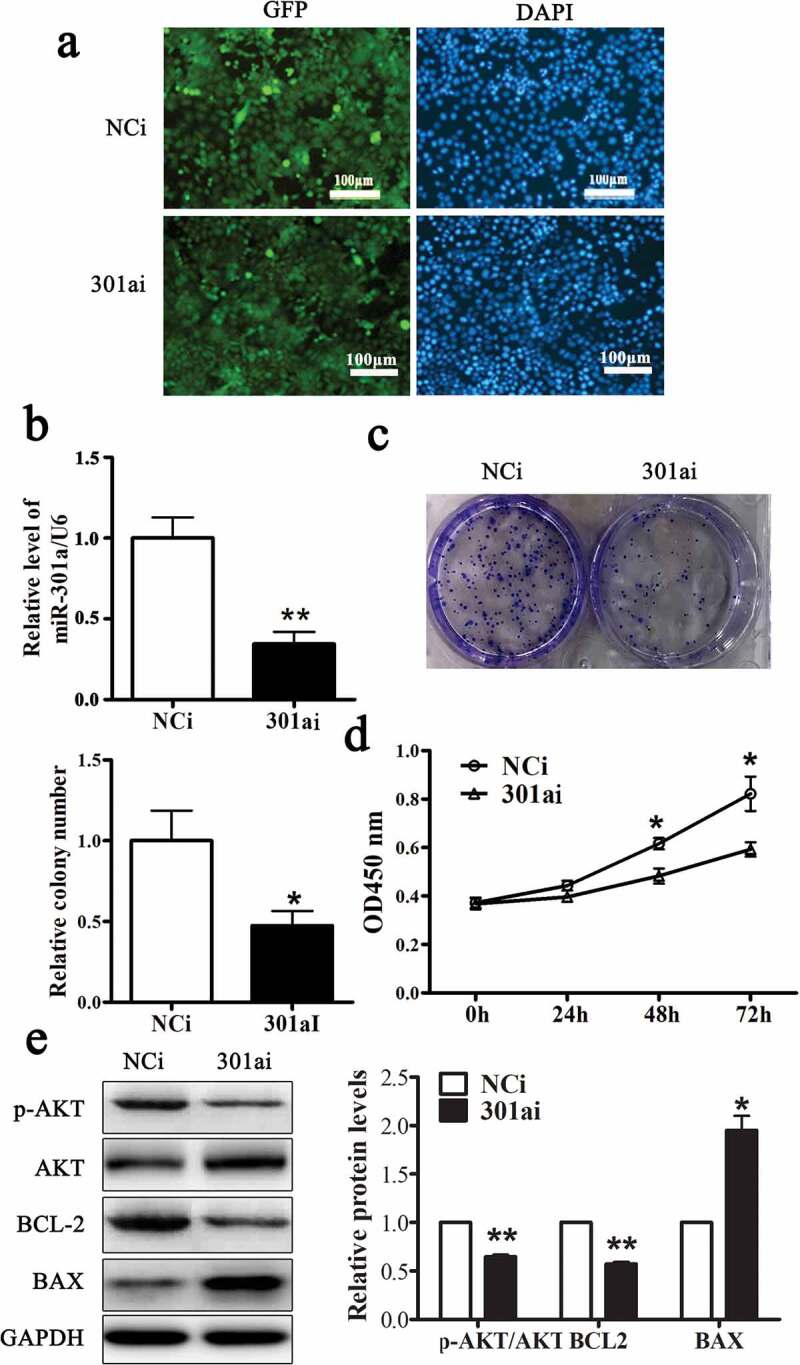
miR-301a-3p inhibits Eca-109 cell proliferation.

Adenovirus vector expressing miR-301a-3p mimic (301am) or negative control mimic (NCm) was transfected into Eca-109 cells to upregulate miR-301a-3p. As Figure 2a showed that more than 80% Eca-109 cell transfected with these two adenovirus vectors expressed GFP. And the relative level of miR-301a-3p/U6 was dramatically raised to fourfold in Eca-109 cell transfected with adenovirus vector expressing miR-301a-3p mimic (Figure 2b). Overexpression miR-301a-3p facilitated the growth of Eca-109 cells (Figure 2c and D) and increased the relative protein levels of p-AKT/AKT and BCL-2 accompanied decreased the relative protein level of BAX (Figure 2e). Moreover, Adenovirus vector expressing miR-301a-3p inhibitor (301ai) or negative control inhibitor (NCi) was transfected into Eca-109 cells to downregulate miR-301a-3p. As Figure 3a showed that more than 80% Eca-109 cell transfected with these two adenovirus vectors expressed GFP. And the relative level of miR-301a-3p/U6 was significantly decreased to approximately 40% (Figure 3b). Down-regulation miR-301a-3p inhibited the growth of Eca-109 cells (Figure 3c and D) and decreased the relative protein levels of p-AKT/AKT and BCL-2 accompanied deceased relative protein level of BAX (Figure 3e). Taken together, these observations showed that miR-301a-3p promoted Eca-109 cell proliferation.

GFP expression in Eca-109 cells transfected with adenovirus vectors expressing miR-301a-3p mimics (301am group) or negative control mimics (NCm group) (A). The relative level of miR-301a-3p/U6 in NCm group and 301am group (B). MTT assay (C) and colony formation assay (D) in NCm group and 301am group. The relative protein levels of p-AKT/AKT, BCL-2 and BAX in NCm group and 301am group (E). *p < 0.05; **p < 0.01 vs NCm group; n = 3.

GFP expression in Eca-109 cells transfected with adenovirus vectors expressing miR-301a-3p inhibitor (301ai group) or negative control inhibitor (NCi group). (A) The relative level of miR-301a-3p/U6 in NCi group and 301ai group (B). MTT assay (C) and colony formation assay (D) in NCi group and 301ai group. The relative protein levels of p-AKT/AKT, BCL-2, and BAX in NCi group and 301ai group (E).*p < 0.05; **p < 0.01 vs NCi group; n = 3.

#### MiR-301a-3p directly targets PTEN

**Figure 4.**
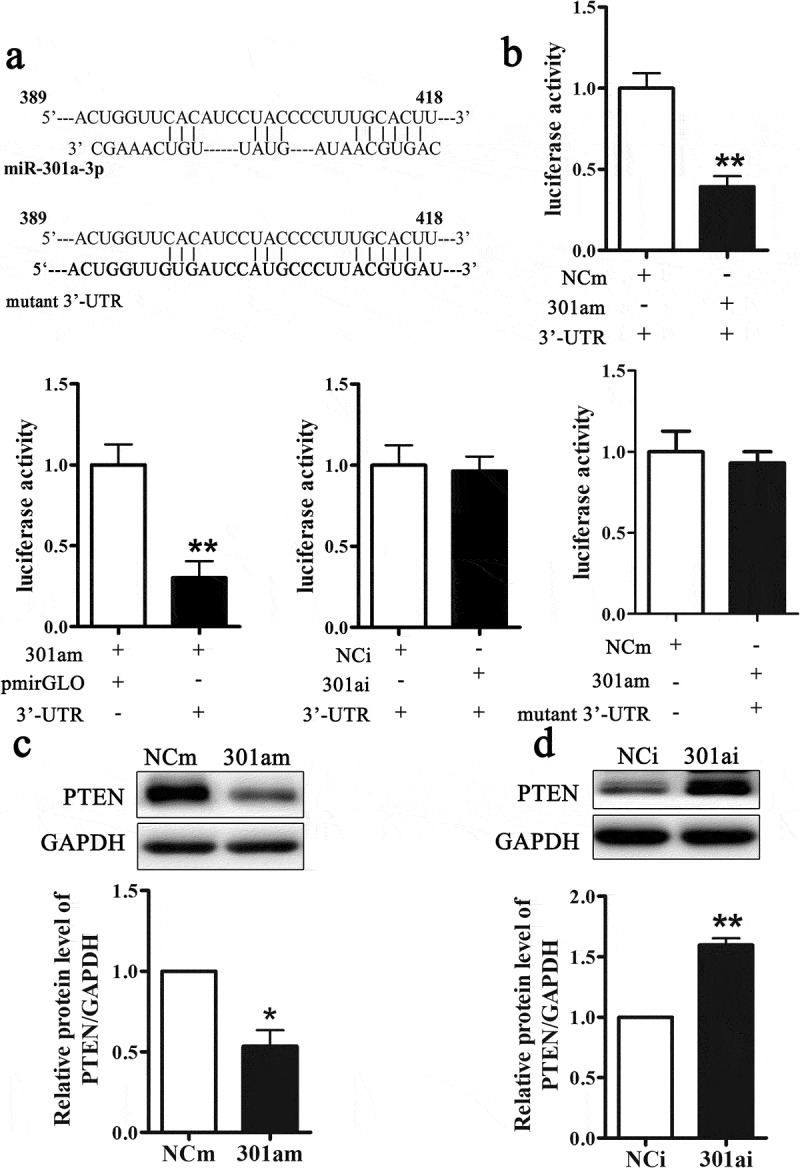
MiR-301a-3p directly targets PTEN.

Previous studies had been verified that microRNAs performed biological functions by modulating target genes. Bioinformatics database TargetScan was used to predict the target genes of miR-301a-3p. As Figure 4 A showed that there was a binding site on 3ʹ-UTR of PTEN mRNA at 398–418 nt. And we mutated the binding site. The fragment of DNA containing 398–418 nt was synthesized and inserted into a pmirGLO vector. As Figure 4b showed that compared with NCm group, transfection with miR-301a-3p mimic inhibited luciferase activity. And compared with control vector group (pmirGLO), transfection with recombinant vector (3ʹUTR) inhibited luciferase activity. But transfection with miR-301a-3p inhibitor did not change luciferase activity. After we mutated the binding site, transfection of the miR-301a-3p mimic could not inhibit luciferase activity (Figure 4b). Next, we determined whether miR-301a-3p could regulate PTEN protein expression. Overexpression miR-301a-3p inhibited the relative protein level of PTEN (Figure 4c), while down-regulation miR-301a-3p increased the relative protein level of PTEN (Figure 4d). Therefore, these results revealed that miR-301a-3p could modulate PTEN protein expression via binding at PTEN mRNA 3ʹ-UTR.

The sequences of a binding site of miR-301a-3p in the PTEN 3ʹ-UTR at 389–418 nt, and the mutated binding site (A). Luciferase activity assay in Eca-109 cells transfected with NCm, miR-301a-3p mimic, NCi or miR-301a-3p inhibitor (B). The relative protein level of PTEN in Eca-109 cells transfected with adenovirus vectors expressing miR-301a-3p mimic (C) or inhibitor (D). *p < 0.05; **p < 0.01 vs NCm group or NCi group; n = 3.

#### The relative protein level of PTEN is reduced in ESCC tissues

**Figure 5.**
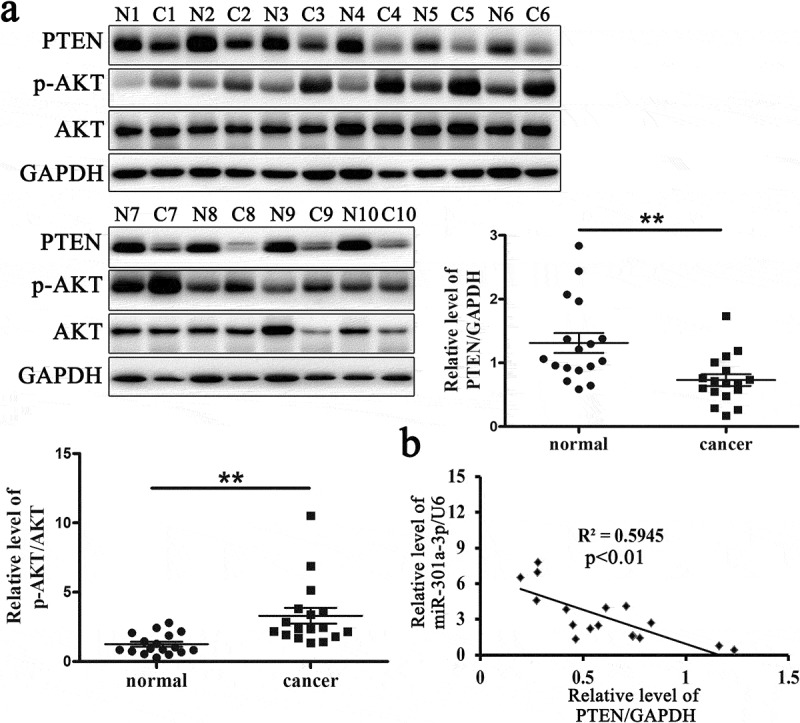
The relative protein level of PTEN is reduced in ESCC tissues.

PTEN is a tumor suppressor gene in various cancers that can negatively regulate the activation of AKT. In our study, we had proved that PTEN was a target of miR-301a-3p. To determine whether miR-301a-3p regulated ECSS cell proliferation via PTEN, we had measured the relative protein level of PTEN and p-AKT/AKT in ESCC tissues and adjacent normal tissues from 17 patients. The relative protein level of PTEN protein was reduced, while the relative protein levels of p-AKT/AKT was raised in ESCC tissues (Figure 5a). Furthermore, the relative level of miR-301a-3p/U6 was negatively correlated with the relative protein level of PTEN in ESCC tissues (Figure 5b). These data indicated that PTEN was related with the pathogenesis of ESCC.

The relative protein levels of PTEN and p-AKT/AKT in ESCC tissues (cancer group) and adjacent normal tissues (normal group) (A). The correlation between the relative level of miR-301a-3p/U6 and the protein level of PTEN in ESCC tissues was analyzed (B). **p < 0.01 vs normal group; n = 17.

#### MiR-301a-3p promotes Eca-109 cell proliferation via targeting PTEN

**Figure 6.**
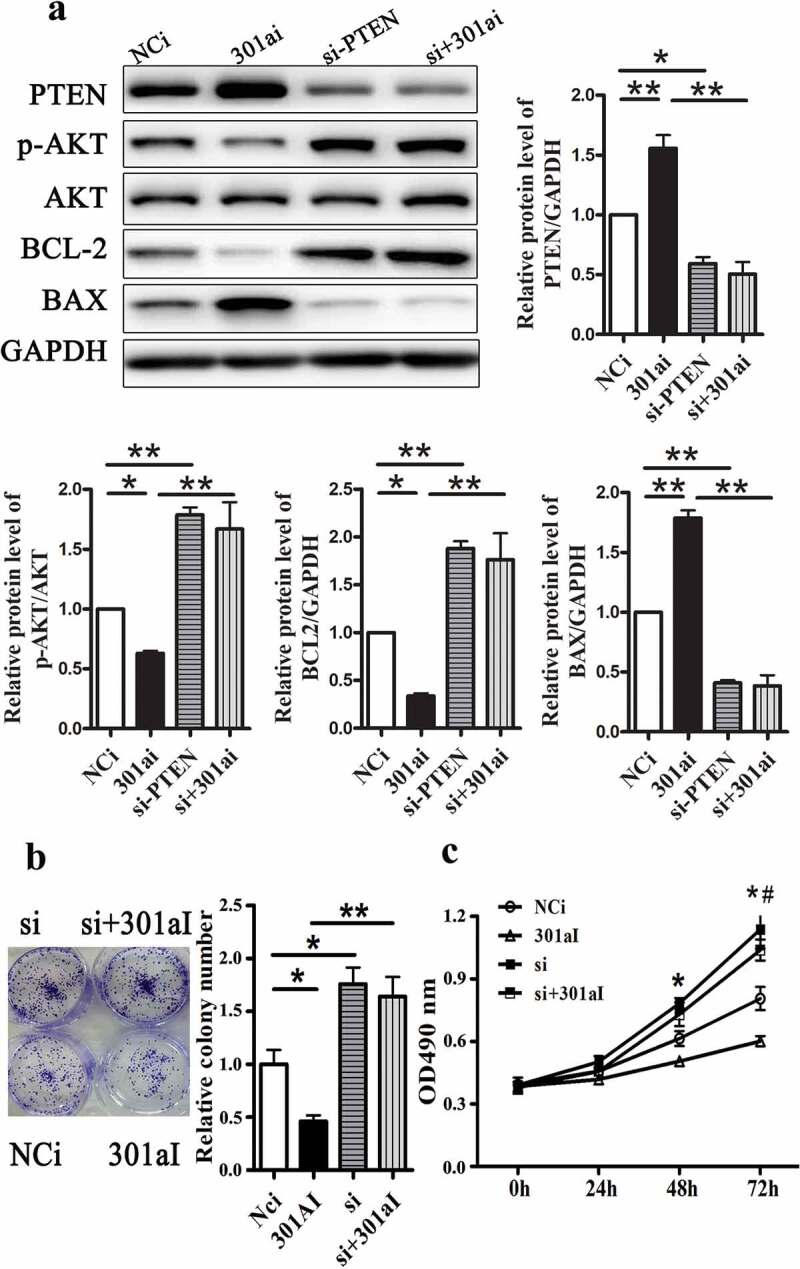
Silencing PTEN reverses inhibited growth of Eca-109 cells induced by miR-301a-3p inhibitor. The relative protein levels of PTEN, p-AKT/AKT, BCL-2 and BAX in Eca-109 cells transfected with PTEN-specific siRNA (si-PTEN group) and miR-301a-3p inhibitor (301ai group) (A). Colony formation assay (B) and MTT assay (C) in Eca-109 cells. *p < 0.05; **p < 0.01 vs NCm group; n = 3; # p < 0.05 vs the 301ai group.

**Figure 7.**
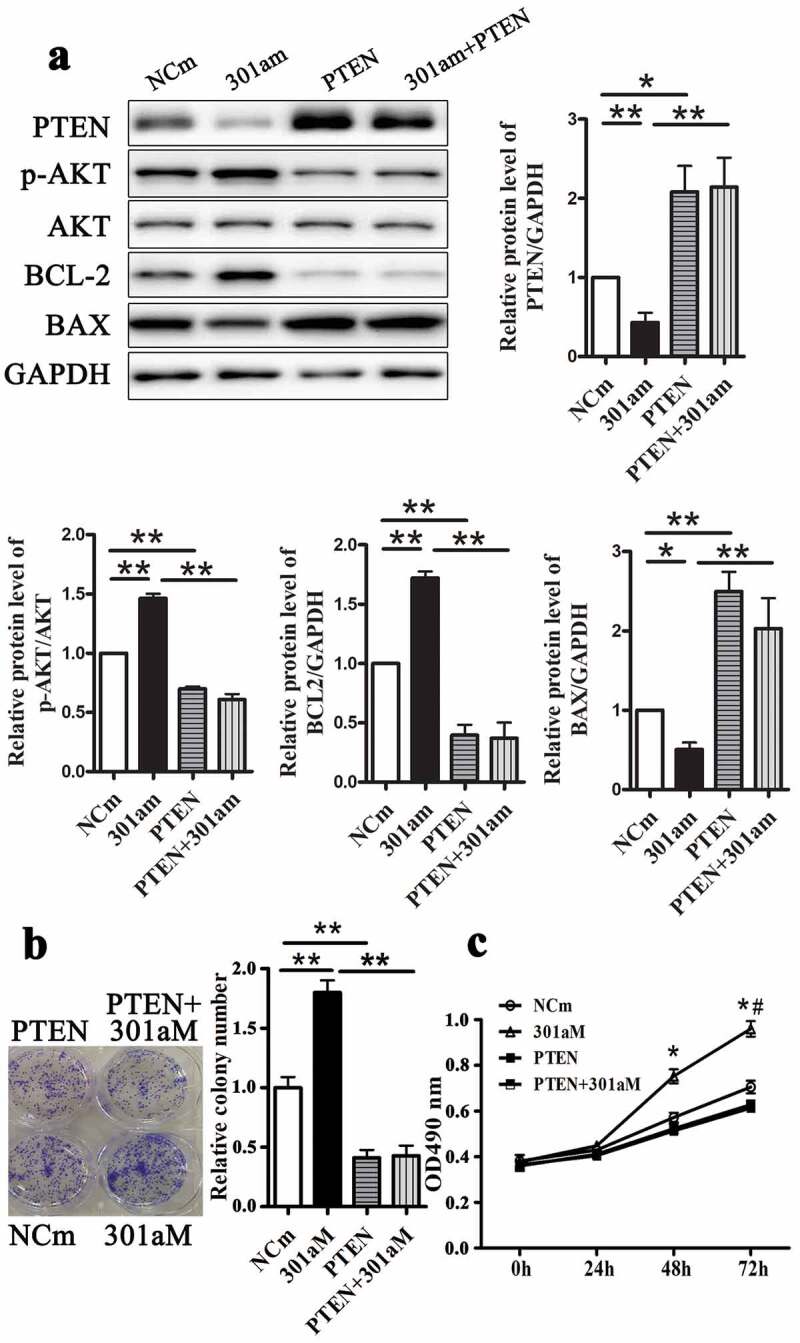
Overexpression PTEN reverse enhanced growth of Eca-109 cells induced by miR-301a-3p mimic. The relative protein levels of PTEN, p-AKT/AKT, BCL-2 and BAX in Eca-109 cells transfected with adenovirus vectors expressing PTEN (PTEN group) and miR-301a-3p mimics (301am group) (A). Colony formation assay (B) and MTT assay (C) in Eca-109 cells. *p < 0.05; **p < 0.01 vs NCi group; n = 3; # p < 0.05 vs the 301am group.

PTEN-specific siRNA was used to silence PTEN protein expression to identify whether miR-301a-3p regulated Eca-109 cell proliferation through PTEN. As Figure 6a showed that inhibition of miR-301a-3p increased the relative protein levels of PTEN and BAX and decreased the relative protein levels of p-AKT/AKT and BCL-2. The proliferation and colony formation of Eca-109 cells were inhibited by transfection with miR-301a-3p inhibitor (Figure 6b and C). However, inhibition of miR-301a-3p did not change PTEN expression and cell proliferation in Eca-109 cells transfected with PTEN-specific siRNA. Next, we up-regulated PTEN expression by transfected with adenovirus vectors expressing PTEN. Overexpression PTEN could reverse the effects of miR-301a-3p mimic on signal pathway and cell proliferation (Figure 7a B and C). Therefore, these observations suggested that miR-301a-3p regulated Eca-109 cell proliferatin via PTEN.

## Discussion

EC leads to a million deaths worldwide every year [14]. ESCC is the main histological subtype of EC in China [2]. At present, Surgery and chemotherapy are the main treatments of ESCC [14,15]. Because many ESCC patients are diagnosed at an advanced stage, the five-year survival rate for patients is still very low. The molecular mechanism of the pathogenesis of ESCC still needs to be explored. In our study, we focused on the role of miR-301a-3p in ESCC cell proliferation.

MicroRNAs can inhibit target gene protein expression by binding to their 3ʹ-UTR, which are related with the pathogenesis of many cancers [16–18]. MiR-301a-3p is a member of miR-301 family that is involved in various cancers, such as pancreatic cancer, osteosarcoma, and breast cancer [12,19,20]. This study indicated that miR-301a-3p was related with the pathogenesis of ESCC. First, we compared the relative levels of miR-301a-3p/U6 between ESCC tissues and adjacent normal tissues. The relative level of miR-301a-3p/U6 was elevated in ESCC tissues. And the relative level of miR-301a-3p/U6 was correlated with TNM stage and tumor size. Next, we determined the effect of miR-301a-3p on Eca-109 cell proliferation. Overexpression miR-301a-3p promoted Eca-109 cell proliferation. Conversely, down-regulation miR-301a-3p restarted Eca-109 cell proliferation. Previous studies had reported that miR-301a-3p directly regulated PTEN protein expression in pancreatic cancer cells and liver cancer cells [12,21]. In consistent, we also found that the relative protein level of PTEN supressed in the ESCC tissues. And the relative level of miR-301a-3p/U6 was negatively correlated with the relative protein level of PTEN in ESCC tissues. Moreover, the results of luciferase assay verified that PTEN was a direct target gene of miR-301a-3p. Then, we determined whether miR-301a-3p regulated ESCC cell proliferation via PTEN. PTEN was a tumor suppressor gene that was frequently deleted or mutated in various tumors, such as breast cancer [22] and lung cancer [23]. PTEN repressed the activation of the PI3K/AKT pathway, which was an important signal pathway related with cell proliferation [24]. The data had proved that miR-301a-3p regulated the proliferation of EC cells by directly targeting PTEN. And miR-301a-3p could also affect the relative protein levels of BCL-2 and BAX. BCL-2 could facilitate the survival of cells and the pathogenesis of cancer [25]. BAX was a pro-apoptotic gene, which was inhibited by BCL-2 [26]. AKT kinase could inhibit the function of FOXOs, which promoted cell apoptosis signaling by regulating BCL-2 family.

## Conclusion

In conclusion, this study suggested that miR-301a-3p regulated the proliferation of EC cells by targeting PTEN, which could be a novel therapeutic target for ESCC.

## Highlights

1. Aberrantly overexpressed of miR-301a-3p leads to EC cancer.
2. The relative level of miR-301a-3p/U6 is correlated with tumor size and TNM stage.
3. Up-regulated miR-301a-3p promotes ESCC cell proliferation via PTEN.
4. MiR-301a-3p may be a novel therapeutic target for ESCC.
